# Optimizing the management of psoriasis in patients with skin of color: A Canadian Delphi consensus

**DOI:** 10.1016/j.jdin.2024.09.015

**Published:** 2024-11-14

**Authors:** Geeta Yadav, Yvette Miller-Monthrope, Jaggi Rao, David N. Adam, Rachel N. Asiniwasis, Parbeer Grewal, Christina Han, Marissa Joseph, Richard G. Langley, Charles W. Lynde, Andrei Metelitsa, Loukia Mitsos, Boluwaji Ogunyemi, Kerri S. Purdy, Maxwell Sauder, Jensen Yeung

**Affiliations:** a Division of Dermatology, Temerty Faculty of Medicine, University of Toronto, Toronto, Ontario, Canada; b FACET Dermatology, Toronto, Ontario, Canada; c Division of Dermatology, Department of Medicine, Women's College Hospital, Toronto, Ontario, Canada; d Division of Dermatology, Department of Medicine, University Health Network, Toronto, Ontario, Canada; e Department of Laboratory Medicine and Pathobiology, Temerty Faculty of Medicine, University of Toronto, Toronto, Ontario, Canada; f Probity Medical Research, Waterloo, Ontario, Canada; g Division of Dermatology, Department of Medicine, University of Alberta, Edmonton, Alberta, Canada; h Baywood Dermatology & CCA Medical Research, Ajax, Ontario, Canada; i Origins Dermatology Centre, Regina, Saskatchewan, Canada; j Division of Dermatology, University of Saskatchewan, Regina, Saskatchewan, Canada; k Rejuvenation Dermatology, Edmonton, Alberta, Canada; l Department of Dermatology and Skin Science, University of British Columbia, Vancouver, British Columbia, Canada; m Department of Pediatrics, Temerty Faculty of Medicine, University of Toronto, Toronto, Ontario, Canada; n Section of Dermatology, Division of Paediatric Medicine, The Hospital for Sick Children, Toronto, Ontario, Canada; o Division of Dermatology, Faculty of Medicine, Dalhousie University, Halifax, Nova Scotia, Canada; p Lynde Institute for Dermatology, Markham, Ontario, Canada; q Beacon Dermatology, Calgary, Alberta, Canada; r Division of Dermatology, Department of Medicine, University of Calgary, Calgary, Alberta, Canada; s Protoderma, Pierrefonds, Quebec, Canada; t Discipline of Medicine, Faculty of Medicine, Memorial University of Newfoundland, St. John's, Newfoundland and Labrador, Canada; u Division of Dermatology, Department of Medicine, Sunnybrook Health Sciences Centre, University of Toronto, Toronto, Ontario, Canada

**Keywords:** Canadian Delphi consensus, disparities in psoriasis management, diversity, equity, psoriasis, skin of color

## Abstract

**Background:**

There is limited evidence on treating psoriasis patients with skin of color (SOC), contributing to disparities in accessing appropriate care for these patients.

**Objectives:**

This study aimed to develop consensus statements defining SOC terminology and addressing needs to optimize the clinical management of psoriasis in patients with SOC.

**Methods:**

Using the modified Delphi methodology 16 Canadian dermatologists with expertise in psoriasis developed consensus statements. Four core faculty members drove the content of the study, and 12 additional panel members were consulted to vote and provide consensus on the content produced by the core faculty. At a final meeting, the full panel revised and voted on the final consensus statements.

**Results:**

The exercise resulted in 11 consensus statements on SOC terminology, as well as 5 primary and 4 secondary statements on clinical presentation and differential diagnosis, and treatment guidelines based on evidence and expert opinion. Four additional consensus statements on current assessment tools and access to care were developed based solely on expert opinion.

**Limitations:**

The available evidence was limited, low quality, and inappropriate for formal quality assessment.

**Conclusions:**

The consensus statements developed in this study may provide valuable guidance to the dermatology community treating psoriasis patients with SOC.

**Capsule Summary**

• There is limited evidence, and hence a lack of guidelines for psoriasis management in people with skin of color.
• In this first-ever consensus study, Canadian dermatologists created a dialogue on skin of color terminology and addressing the need to optimize the clinical management of psoriasis patients with skin of color.

## Introduction

Chronic plaque psoriasis is a common skin condition caused by a dysregulated immune system, traditionally characterized by erythematous, indurated, scaly, pruritic, and sometimes painful plaques, and affects about one million Canadians.^1^^,^^2^ Skin color is an important determinant of the pathophysiology, disease presentation, treatment, and epidemiology of dermatological conditions.^3^, ^4^, ^5^ However, there are limited data and understanding of psoriasis in skin of color (SOC) patients. SOC patients have unmet needs and challenges to accessing appropriate care and timely diagnosis.^3^^,^^4^^,^^6^

The presentation of psoriasis may differ for patients with SOC.^7^, ^8^, ^9^, ^10^ This can contribute to underdiagnosis, misdiagnosis, suboptimal treatment, and reduced access to care in this subset of patients.^7^^,^^8^^,^^11^ For example, psoriasis in SOC patients presents more with violaceous, grey, and/or brown hues rather than the more obvious erythema that is classically taught.^12^^,^^13^ Additionally, psoriasis in patients with SOC have higher rates of pigmentation, thicker plaques, more scaling, greater body area involvement, and more severe scalp involvement.^8^ Such variations in the clinical presentation can contribute to challenges in diagnosing psoriasis in SOC people.^14^

Psoriasis patients with SOC are less likely to be treated with biologics and there is a lack of awareness of available therapies, including biologics amongst these patients.^15^, ^16^, ^17^, ^18^ Since racial and ethnic populations are frequently under-represented in clinical trials, and there are no specific validated metrics for this population, data on the efficacy of biologic therapies in non-white psoriasis patients is insufficient and/or may be subject to interpretation bias.^19^, ^20^, ^21^

While there is growing awareness and understanding of the significant unmet need in patients with SOC within the dermatology community, clinical trial data to guide psoriasis management is lacking. Nonetheless, the existing literature and anecdotal evidence gained through experience in clinical practice may be leveraged to promote new standards and guidelines in several areas pertinent to SOC patients through systematic consensus development.

This exercise aims to develop consensus statements integrating the best available evidence on optimized diagnosis and management of psoriasis in SOC patients with the goal of providing guidance to the broader dermatology community.

## Methods

### Study design

**Fig 1:**
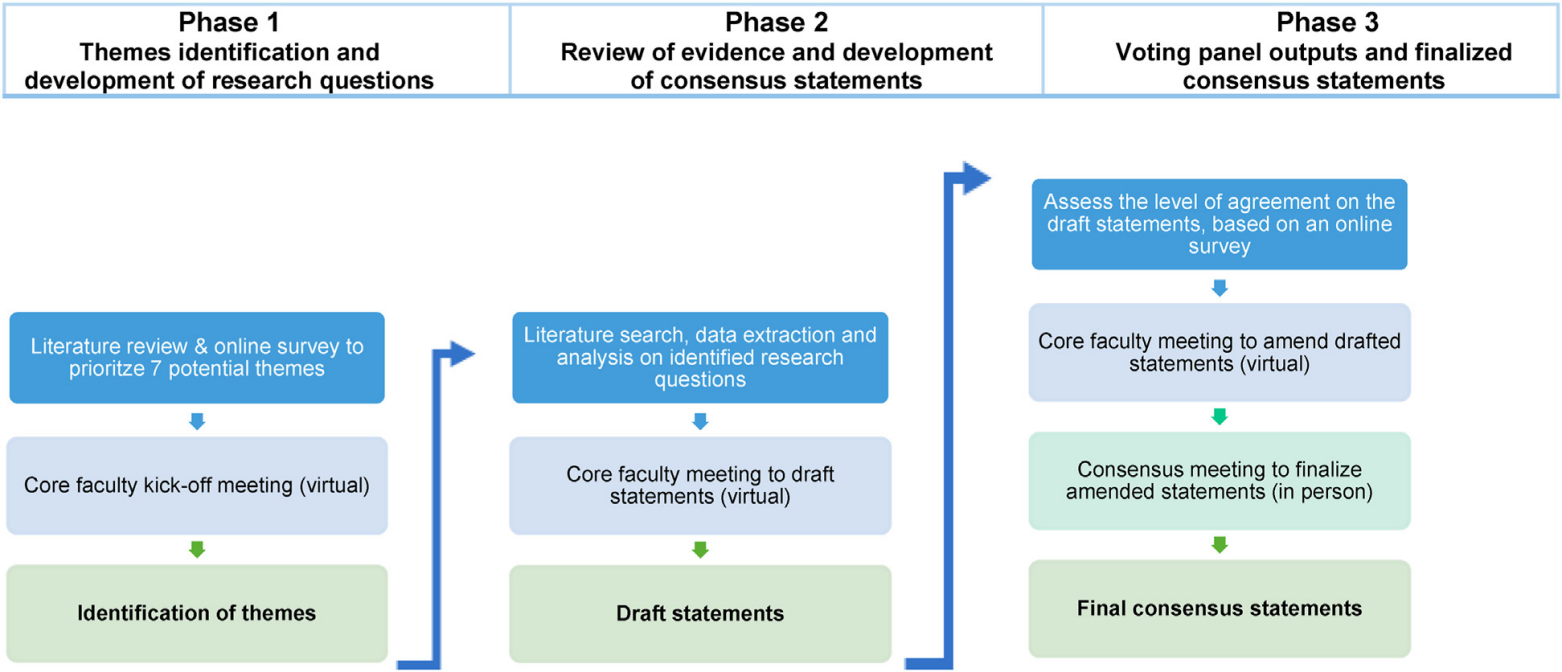
Modified Delphi methodology to build consensus on the “skin of color terminology” and “optimizing the clinical management of psoriasis in patients with SOC”. The national panel and the core faculty voted on themes and draft statements online, and the final consensus statements in-person.

This study used a modified Delphi method,^22^ which included a consensus meeting for expert interaction in the final phase of the consensus-building process. The objective was to produce statements defining SOC terminology and optimizing the clinical management of psoriasis in SOC patients (Fig 1).

A geographically and ethnically diverse group of 16 dermatologists with expertise in psoriasis and skin diversity were recruited from across Canada. Initially, a core faculty (G. Y., J. R., J. Y., and Y. M. M.) was invited to develop the program content. Subsequently, a larger national panel (A. M., B. O., C. L., C. H., D. A., K. P., L. M., M. J., M. S., P. G., R. A., and R. L.) was consulted to provide consensus on the content produced by the core faculty. The core faculty and national panel revised and voted on the consensus statements at a final meeting.

As this was a noninterventional study, no Institutional Review Board approval was required.

## Results

The Delphi consensus on optimizing the clinical management of psoriasis in SOC patients took place between May 2, 2022, (virtual kick-off meeting) and November 18, 2022 (in-person meeting to finalize the consensus statements).

### Baseline demographics and clinical experience of the consensus panel

Thirty-eight percent of the participants were female. The panel had diverse geographic representation, with 44% of participants practicing in Ontario, 19% in Alberta, 13% in Nova Scotia, and 6% in each of British Columbia, Newfoundland, Saskatchewan, and Quebec.

The panel had a broad range of clinical experience; 10% had 1-5 years; 30% had 6-10 years, 30% had 11-20 years, 10% had 21-30 years, and 20% had more than 30 years in dermatology practice.

### Phase 1: Themes identification and development of research questions

Seven potential themes were initially generated following a literature review. The 16 experts prioritized the seven themes via an online survey (Table I). They voted “yes” if they approved of a theme and “no” if they disapproved. If the response was “yes,” they answered 3 related questions: (1) What are your main considerations on this theme? (2) What are your opinions on the matter? and (3) Are there any other aspects to be considered on this theme? They also ranked all themes in order of priority (Table I).

**Table I:** Themes prioritization (online survey)

Themes prioritized by the voting consensus panel members	Percentage (%) of theme approval
○ Skin of color terminology	100
○ Clinical presentation and differential diagnosis	100
○ Guiding principles of treatment	100
○ Patient education	93
○ Access to care	79
○ Adequacy of current assessment tools	71
○ Others	36

The 3 themes that received unanimous approval by the expert panel, SOC terminology, clinical presentation and differential diagnosis, and guiding principles of treatment, were selected by the core faculty for a targeted literature search (Supplementary Appendix, Figs 1 and 2, available via Mendeley at https://doi.org/10.17632/kdzsg62whg.1). Two additional themes, access to care and adequacy of current assessment tools, were also selected based on the expert panel’s responses to the survey questions.

### Phase 2: Review of evidence and development of consensus statements

Targeted literature searches were conducted by a medical librarian. The core faculty drafted consensus statements following evidence review and a virtual workshop.

The drafted statements were grouped into 3 main domains deemed highest priority by the expert panel in the Phase 1 survey: (1) “SOC terminology”, (2) “Clinical presentation, differential diagnosis, and guiding principles of treatment”, and (3) “Additional themes (adequacy of current assessment tools and access to care).”

### Phase 3: Voting panel outputs and finalized consensus statements

In a second online survey, all experts voted on their level of agreement on the draft consensus statements. Each statement was rated on a scale of 1-10, with 10 meaning “strongly agree.” A rating of 5 or less required specifying what aspect(s) of the statement needed revising. A statement rated 6 to 9 required further elaboration on how the statement needed to be worded to receive a 10 rating.

The final round to refine the amended consensus statements and capture the final level of agreement was conducted during an in-person meeting. The experts finalized the statements during 2 concurrent breakout sessions and then as a whole group. Electronic touchpads were used to vote on final agreements using a scale of 1-5 (5 - “strongly agree,” 4- “agree,” 3- “neither agree nor disagree,” 2- “disagree,” and 1- “strongly disagree”). Consensus agreement was defined as ≥75% panelists voting an agreement score of 4 or 5 for a given statement. Simple majority agreement and disagreement were defined as >50% and <50% of panelists voting with an agreement score of 4 or 5, respectively. When an agreement threshold of 75% was not reached in the first round of voting, a second and final round of voting was conducted after statement revision. A simple majority agreement was also valid to establish a consensus.

#### SOC terminology

Two research questions were examined using information derived from a systematic, targeted literature search: (1) Are the existing classification systems and terminology effective in describing SOC in dermatology? and (2) Is the existing terminology effective in describing SOC?

The literature search for each research question identified 35 and 11 publications, respectively, that were used as a basis to develop consensus statements on SOC terminology. Based on output from the literature review and discussions at the virtual workshop, the core faculty initially developed 12 draft consensus statements for this theme, which all experts reviewed for agreement (Supplementary Table I, available via Mendeley at https://doi.org/10.17632/kdzsg62whg.1). Based on the feedback, the core faculty amended these, to yield a total of 11 draft consensus statements. Subsequently, 10 statements met the 75% agreement threshold at the in-person consensus meeting, and one statement met the simple majority agreement threshold (Table II).

**Table II:** Final skin of color terminology consensus statements

	Final consensus statements (16 responders)	Level of agreement (%)
1	There is currently no standard nomenclature to comprehensively describe the spectrum of all skin color. To represent cutaneous diversity, we must commit to the development of a classification system inclusive of all skin colors.	96.2
2	The use of geographic origin, race, or existing skin classification scales, such as the Fitzpatrick phototypes, as proxies for skin color are inadequate as objective measures.	100
3	Objective scientific tools, such as colorimeters and spectrophotometers, can provide reproducible and quantitative measurements of skin color without biased and inaccurate reporting associated with subjective classifications. Although their current clinical use remains limited, measurements made with these instruments could provide physicians with a unified language regarding skin color.	96.2
4	There is a need to develop comprehensive skin color image databases to reflect the diversity of patients and disease presentations.	100
5	The concept of “race” has no scientific justification.	92.6
6	Racial, ethnic, and geographical terms are unrelated to skin traits and should be avoided.	88.8
7	The term “skin of color” is used for dermatological purposes because it relates to the descriptive use of clinical presentations and biological traits of pigmented skin.	96.2
8	Descriptive vocabulary must be adopted to be inclusive and culturally appropriate reflecting cutaneous diversity.	97.6
9	It is important to ensure proper representation of patients with skin of color in all dermatology educational materials.	100
10	It is important to promote education to, and utilization of skin of color concepts with educators, researchers, healthcare providers, and the general public.	100
11	With innovation, dermatology has the opportunity to lead the effort against biases associated with skin color assessment, so that new technology can be evaluated and validated for future use by the medical community. (**Simple majority agreement**).	70

The consensus statements indicated that standard nomenclature to comprehensively describe the spectrum of all skin colors was lacking in the medical literature. Further, parameters such as geographic origin, race, or existing skin classification scales (eg, Fitzpatrick phototype) were commonly used as surrogates for skin color but are inadequate and should be avoided. Lastly, the experts concluded that there is a need to develop a novel classification system inclusive of all skin colors and comprehensive image databases of different skin colors and/or image databases with skin disease in patients with varied skin colors.

#### Clinical presentation, differential diagnosis, and guiding principles of treatment

Two research questions were examined based on output from a systematic, targeted literature search: (1) What is the clinical presentation, time to diagnosis, and treatment response in people with SOC who have psoriasis? and (2) What are the treatment considerations in people with SOC who have psoriasis?

A literature search identified 35 and 17 publications for each research question, respectively, that were used as a basis to develop consensus statements on clinical presentation, differential diagnosis, and guiding principles of treatment.

Based on the findings from the literature review and discussions at the virtual workshop, the core faculty initially developed 10 consensus statements, which all experts reviewed for agreement (Supplementary Table II, available via Mendeley at https://doi.org/10.17632/kdzsg62whg.1). Based on further discussion, the core faculty amended them to a total of 8 statements. Finally, at the in-person meeting, these were further amended to produce 5 main statements and 4 substatements. Subsequently, all met the 75% agreement threshold in the final consensus voting process (Table III).

**Table III:** Final consensus statements: Clinical presentation, differential diagnosis, and guiding principles of treatment

	Final consensus statements (16 responders)	Level of agreement (%)
1	Special considerations are needed when assessing psoriasis in patients with skin of color, as clinical presentation may be different than in white skin.	96.2
2	The scalp is a challenging site to treat. Hair texture, care patterns, hair washing frequency, head coverings, and patient preferences should be considered when developing a management plan to treat scalp psoriasis.	96.2
3	Pigmentary alteration disproportionately impacts patients with skin of color and may negatively affect quality of life.	100
4	Pigmentary alteration should be considered in the management of psoriasis: a Consideration should be given when prescribing topical therapies in patients with skin of color as they may contribute to pigmentary alteration. b Phototherapy in individuals with skin of color may temporarily darken the skin which may not be acceptable for some patients. c Early initiation of systemic therapies should be considered, when appropriate, in patients with skin of color to minimize the sequelae of disease related pigmentary alteration. d Proper education, counselling, additional studies, and treatment guidelines are needed to prevent and manage dyspigmentation.	93.8 96.2 87.6 90
5	There is limited evidence that the efficacy of systemic therapies varies in different populations, including skin of color. More research is required.	93.8

The panel of experts agreed that assessing psoriasis in SOC patients requires special attention, as the clinical presentation may be different compared to white skin. The panel also agreed that pigmentary alteration must be considered in managing psoriasis in SOC patients, as it disproportionately impacts these patients and may profoundly affect their quality of life. Therefore, special considerations were added regarding phototherapy and topical therapies, as these modalities may contribute to pigmentary alteration in SOC patients. Emphasis was placed on early initiation of effective systemic therapy to minimize pigmentary sequelae.

#### Additional themes

Two additional themes of interest were identified: (1) How well do current assessment tools (example: Psoriasis Area and Severity Index, Investigator’s Global Assessment, Dermatology Life Quality Index, Physician’s Global Assessment) capture pretreatment and post-treatment disease status? and (2) How can dermatologists improve access to care for psoriasis patients with SOC?

The core faculty developed 3 statements for the additional themes, which all experts later reviewed (Supplementary Table III, available via Mendeley at https://doi.org/10.17632/kdzsg62whg.1). No further amendments to these statements were made based on feedback from the experts at this stage. However, at the final in-person meeting, a fourth consensus statement was added, and all met the 75% agreement threshold upon subsequent voting (Table IV).

**Table IV:** Final consensus statements on additional themes

	Final consensus statements (16 responders)	Level of agreement (%)
1	Psoriasis clinical trials predominantly enroll white individuals which limits applicability of results to patients with skin of color. Future studies should have more equitable representation.	100
2	There is a need for assessment tools that do not rely on erythema as a marker for inflammation.	77.6
3	It is crucial that dermatologists and other stakeholders in the health care system recognize and act on health disparities affecting people with psoriasis and skin of color to address health inequities.	81.2
4	Indigenous populations have inequitable access to dermatologic care and suffer health disparities. Efforts towards reconciliation in dermatology should include improved education, engagement, and representation.	91.2

The experts agreed on an unmet need for a psoriasis assessment tool(s) that does not weigh on grading erythema as the key marker of inflammation. They also acknowledged the substantial lack of SOC patients in clinical trials. Patients recruited into psoriasis trials are predominantly white, limiting the applicability of the results to SOC patients. Of note, the ongoing VISIBLE study (NCT05272150) aims to address this gap and is dedicated to evaluating psoriasis and treatment outcomes in SOC patients.^23^

## General discussion

The current work used a modified Delphi methodology to develop consensus statements defining SOC terminology and addressing needs to optimize the clinical management of psoriasis in SOC patients. This is the first study conducted focusing on the unmet needs of the SOC patient population in Canada.

A geographically diverse group of 16 dermatologists with expertise in the management of psoriasis patients with SOC from across Canada participated in this study. The methodology implemented, with targeted literature reviews, multiple online surveys, and virtual and live meetings, facilitated development of an iteratively modelled and precise set of consensus statements. The SOC terminology statements can be applied for use by the broader dermatology community and potentially be extended to help guide the management of other skin conditions in SOC patients.

The core faculty selected 3 themes (SOC terminology, clinical presentation and differential diagnosis, and guiding principles of treatment) to develop consensus statements based on evidence and expert opinion. Two additional themes were identified (adequacy of current assessment tools and access to care) to develop consensus statements based solely on expert opinion.

Evidence shows disparities in accessing appropriate care for dermatological conditions like psoriasis for people with SOC.^3^ Often misdiagnosed, psoriasis can significantly impact the quality of life of patients with SOC. Further, psoriasis patients with SOC are frequently under-represented in research studies and clinical trials.^16^^,^^17^ Given the lack of data and evidence, it can be more challenging for dermatologists to provide adequate care for patients with SOC. It is, therefore, important to create a scientific dialogue and consensus statements around identified gaps based on clinical experience in managing psoriasis in SOC patients.

The experts unanimously agreed that the existing skin classification systems, such as the Fitzpatrick skin phototype scale, are inadequate for characterizing SOC. The Fitzpatrick phototype scale was developed to describe how the skin responds to ultraviolet radiation exposure and does not identify skin color. However, all experts agreed that despite its shortcomings, the Fitzpatrick skin phototype scale is widely used among dermatologists and referenced in medical textbooks and hence should be included in the consensus statements. Nonetheless, there remains a need for a classification system that is more inclusive of all skin colors and for tools that can provide reproducible, quantitative measurements without the bias and inaccuracy of subjective classifications. Minimizing biases associated with skin color assessment is essential for advancing the field of dermatology. New technological innovations can change how dermatologists assess skin and allow for better classification of skin color. While reliable, objective tools such as colorimetry and spectrophotometry are available and could provide physicians with a standardized approach for assessing skin color, their use in clinical practice is currently limited.

Postinflammatory pigmentary alteration disproportionately affects SOC patients and can negatively impact their quality of life. Therefore, the risk of pigmentary alteration must be minimized when creating a treatment plan for SOC patients and could be considered as another measure of efficacy for therapies. Education, counseling, additional studies, and treatment guidelines are needed to better support patients in order to minimize such risks.

The approach to managing psoriasis involving sensitive/high impact areas represents another gap. The scalp is a particularly challenging area to treat. When treating scalp psoriasis in SOC patients, the vehicle used for topical therapies must be compatible with the patient's preference, hair texture, frequency of hair washing, and hairstyle methods. Such considerations are important to enhance treatment adherence and ultimately treatment outcomes.

Several other gaps were identified. The experts recognized that racial, ethnic, and geographical terms are unrelated to skin traits and that race does not have scientific justification. They uniformly agreed that a comprehensive SOC disease image database reflecting the diversity of patients and disease presentations must be developed. All dermatology education materials must also ensure proper and diverse representation of SOC patients. The experts also acknowledged the importance of psoriasis clinical trials enrolling more non-white patients to provide more data to better inform psoriasis management in SOC patients. Dermatologists and stakeholders must recognize the disparities in psoriasis management in SOC patients and take steps to close the gaps through improved education, engagement, and representation. Lastly, specific to the Canadian population, an additional consensus statement was added, highlighting the need to recognize and address barriers and health inequities faced by Indigenous peoples, particularly those living in rural and remote communities when trying to access dermatologic care.

The main limitation of this study was the lack of sufficient evidence for a formal quality rating when performing targeted literature searches. This further emphasizes the importance of more research studies and clinical trials of psoriasis, including more SOC patients.

## Conclusions

This study provides valuable guidance to the broader dermatology community on the optimized clinical management of psoriasis in SOC patients. It also highlights the gaps in evidence and calls for better assessment tools and more research to help bring about improved psoriasis management in this patient population.

## Conflicts of interest

Dr Yadav received honoraria from AbbVie, Amgen, Aralez, Arcutis, Bausch Health, Bioderma, BI, BMS, Byrdie, Galderma, Incyte, Janssen, Johnson & Johnson, Leo, Eli Lilly, L’Oreal, Medexus, Novartis, Pfizer, Sanofi-Regeneron, Sun Pharma, and UCB for advisory board and speaker services and for her participation in this study. Dr Rao received honoraria from Janssen for advisory board and speaker services and for his participation in this study. Dr Yeung received honoraria from AbbVie, Amgen, Anacor, Astellas, Arcutis, Bausche, Baxalta, Boehringer Ingelheim, BMS, Celgene, Centocor, Coherus, Dermira, Eli Lilly, Forward, Galderma, Incyte, Janssen, Leo, Medimmune, Merck, Novartis, Pfizer, Regeneron, Roche, Sanofi Genzyme, Sun Pharma, Takeda, UCB, and Xenon for advisory board and speaker services and for his participation in this study. Dr Miller-Monthrope received honoraria from AbbVie, Bausch Health, Galderma, Incyte, Janssen, Novartis, Sanofi-Regeron, Sun Pharma, and UCB for advisory board and speaker services and for her participation in this study. Dr Metelitsa received honoraria from Abbvie, Amgen, Bausch, Boehringer Ingelheim, BMS, Celgene, Eli Lilly, Galderma, Incyte, Janssen, Leo, Norvartis, Pfizer, Sanofi Genzyme, Sun Pharma, and UCB for advisory board and speaker services and for his participation in this study. Dr Ogunyemi received honoraria from AbbVie, Janssen, Novartis, Sun Pharma, Pfizer and UCB for advisory board and speaker services and for his participation in this study. Dr Lynde received honoraria from AbbVie, Amgen, Aralez, Arcutis, Bausch Health, Bayer, Boehringer Ingelheim, Bristol Myers Squibb, Celgene, Cipher, Dermavant, Eli Lilly, Fresnius Kabi, Galderma, GSK, Incyte, Innovaderm, Intega Skin, Janssen, Kyowa Kirin, La Roche Posay, LEO Pharma, L'Oreal, Medexus, MedX, Merck, Novartis, P&G, Pediapharm, Pfizer, Regeneron, Roche, Sanofi Genzyme, Sandoz, Sentrex, SunPharma, TEVA, Tribute, UCB, Valeant, Viatris, Volo Health for advisory board and speaker services and for his participation in this study. Dr Han received honoraria from Abbvie, Amgen, Arcutis, Bausch Health, Celgene, Galderma, Janssen, Leo Pharma, Eli Lilly, Novartis, Sanofi Genzyme, Sun Pharma, UCB, and XYON for advisory board and speaker services and for her participation in this study. Dr Adam received honoraria from AbbVie, Arcutis, Amgen, Actelion, Arcutis, Bausch Health, Boehringer Ingelheim, BMS, Celgene, Coherus, Dermira, Dermavant, Eli Lilly, Galderma, Incyte, Janssen, Leo Pharma, Merck, Novartis, Pfizer, Reistone, Regeneron, Sanofi Genzyme, Sun Pharma and UCB for advisory board and speaker services and for his participation in this study. Dr Purdy received honoraria from Abbvie, Amgen, Arcutis, Boerhinger-ingleheim, BMS, Bausch Health, Eli Lilly, Incyte, Janssen, Leo, Novartis, Pfizer, Recordati, Sanofi, Sun Pharma, and UCB for advisory board and speaker services and for her participation in this study. Dr Mitsos received honoraria from AbbVie, Amgen, Aralez, Arcutis, Bausch Health, Bioderma, BMS, Galderma, Janssen, Johnson & Johnson, Leo, Eli Lilly, L’Oreal, Medexus, Novartis, Pfizer, Sanofi-Regeneron, Sun Pharma, and UCB for advisory board and speaker services and for her participation in this study. Dr Joseph received honoraria from Abbvie, Amgen, Arcutis, Bausch, Boehringer Ingelheim, BMS, Celgene, Eli Lilly, Galderma, Incyte, Janssen, Leo, L’Oreal, Norvartis, Pfizer, Sanofi Genzyme, Sun Pharma, and UCB for advisory board and speaker services and for her participation in this study. Dr Sauder received honoraria from Amgen, AbbVie, Bausch Health, Boehringer Ingelheim, Bristol-Myers-Squibb, Janssen, LEO Pharmaceuticals, Novartis, Sun Pharmaceuticals, UCB Canada, and Viatris for advisory board and speaker services and for his participation in this study. Dr Grewal received honoraria from AbbVie, Amgen, Anacor, Arcutis, Arena Pharmaceuticals, Avillion, Bausch Health/Valeant, Boehringer Ingelheim, BMS, Celgene, Cipher, Dermavant, Dermira, Eli Lilly, Galderma, GSK, Incyte, Innovaderm, J&J/Janssen, Leo Pharma, Med Plan, Meiji Seika Pharma, Merck, Novartis, Pfizer, Regeneron, Sanofi-Aventis/Genzyme, Sandoz, Sun Pharmaceuticals, Takeda, UCB, and Vitae for advisory board and speaker services and for his participation in this study. Dr Asiniwasis received honoraria from Abbvie, Arcutis, Bausch, Boehringer-Ingleheim, Chronicle Companies, Galderma, Incyte, Janssen, Leo, Eli Lilly, L'Oreal, Medexus, Novartis, Pfizer, Sanofi, Sun Pharma, UCB, and WoundPedia for advisory board and speaker services and for her participation in this study. Dr Langley received honoraria from AbbVie, Amgen, Bausch Health, BI, BMS, Janssen, Leo, Eli Lilly, Novartis, Pfizer, Sanofi-Regeneron, Sun Pharma, and UCB for advisory board and speaker services and for his participation in this study.
